# Epigenetic editing and epi-drugs: a combination strategy to simultaneously target KDM4 as a novel anticancer approach

**DOI:** 10.1186/s13148-025-01913-0

**Published:** 2025-06-19

**Authors:** Federica Sarno, Jim J. Jacob, Roos E. Eilers, Angela Nebbioso, Lucia Altucci, Marianne G. Rots

**Affiliations:** 1 Epigenetic Editing Research Group, Department of Pathology and Medical Biology, University of Groningen, University Medical Center Groningen, Hanzeplein 1, 9713 GZ Groningen, The Netherlands; 2 Dipartimento Di Medicina Di Precisione, Università Degli Studi Della Campania “Luigi Vanvitelli” Napoli, Naples, Italy; 3 Biogem, Molecular Biology and Genetics Research Institute, Ariano Irpino, Italy; 4 Medical Epigenetics Program, Azienda Ospedaliera Universitaria “Luigi Vanvitelli”, Naples, Italy

**Keywords:** Epigenetic editing, CRISPR/dCas9, KDM4, Epi-drugs, QC6352, JIB-04

## Abstract

KDM4-A/B/C, preferentially demethylating di- and tri-methylated lysine 9 on histone H3, are overexpressed in cancers and considered interesting therapeutic targets. Consequently, KDM4 inhibitors have been developed to block their enzymatic activity. However, the potential lack of specificity of such small molecules (epi-drugs) may contribute to dose-limiting toxicities. In the pursuit of more specific interventions, epigenetic editing (epi-editing) has emerged as a powerful tool to modulate gene expression by modifying the epigenetic profile of specific genomic locations. The recently developed CRISPRoff (dCas9 fused to DNMT3A/3L and KRAB), guided by sgRNAs, is successfully used for gene repression by introducing methylation of DNA and (indirectly) of histones at the targeted genomic region. We propose that combining epi-editing (here to prevent the expression of KDM4) with epi-drugs (to inhibit the KDM4 protein activity) may represent a novel path for synergistic anticancer effects through simultaneous inhibition of gene expression and protein activity. Upon validating the downregulation of KDM4A in HEK293T cells through epi-editing, we demonstrated its repression in colon, breast and hepatocellular carcinomas which was effective in preventing (breast, MCF7) or inhibiting (colon, HCT116) cancer cell growth. Anticancer effect was also confirmed for these cell lines using the KDM4 inhibitor QC6352. In parallel, our studies demonstrate a previously unnoticed increase in the expression of KDM4-A/B/C genes following the inhibition of protein activity using the pan-KDM4 inhibitors QC6352 and JIB-04. Importantly, this induction of gene expression was fully prevented or even further inhibited by epi-editing. Then, we assessed the efficacy of our dual-targeted silencing approach in cancer cells and demonstrated that the inhibition in cancer cell growth by epi-drug or epigenetic editing could be further improved by combining the treatments. Building upon these findings, we introduce a novel, potentially synergistic, therapeutic strategy that combines epi-drug administration with epi-editing. This innovative approach aims to reduce drug toxicity and the potential development of resistance by preventing drug-induced upregulation of target enzyme expression, thereby further increasing anticancer effects.

**Supplementary Information:**

The online version contains supplementary material available at 10.1186/s13148-025-01913-0.

## Introduction

Epigenetic reprogramming frequently occurs in human diseases, including cancers [1–4], and often stems from dysregulated activity of epigenetic proteins [2]. Consequently, chromatin regulators have emerged as compelling targets for therapeutic intervention. Currently, nine inhibitors of epigenetic enzymes, so-called epi-drugs, have received clinical approval for cancer treatment, primarily for treating hematological malignancies [5, 6]. However, expanding their application to solid tumors encounters challenges including a short half-life, requiring high dosage over a long period of time to translate into therapeutic effects, dose-limiting toxicities [7, 8] and development of resistance [9]. Ongoing investigations involve the exploration of novel epi-drugs, either individually or in combination with standard chemotherapeutic regimens. It is well established that drug resistance poses a significant challenge for anticancer agents, with compensatory upregulation of the enzymatic target following pharmacological inhibition posing a considerable drawback in the landscape of conventional chemotherapy [10]. However, these mechanisms are not yet established in the context of epi-drugs [11].

As candidates for epi-drugs, lysine demethylase 4 (KDM4) enzymes have received interest because of their overexpression in various cancers including breast, colon, lung, prostate and ovary [12, 13]. KDM4 family members catalyze the removal of K9 and K36 methylation marks from histone H3. KDM4-A/B/C are the most studied members and share over 50% of protein structure [12]. Various insights into the oncogenic mechanisms have been described, mostly for KDM4A [14–17]. KDM4A can, for example, form a complex with ERα, inducing ERα-mediated transcription in breast cancer [18]. Indeed, KDM4A knockdown in MCF-7 breast cancer cells resulted in the reduction of oncogenic properties [19]. In colon cancer, DNA damage induced by adriamycin leads to the suppression of p21 due to the recruitment of KDM4A and p53 to its promoter. Silencing of KDM4A indeed increased the expression of p21 and pro-apoptotic genes while downregulating the expression of the antiapoptotic Bcl-2 [17]. Also, for KDM4B and KDM4C increased expression has been reported in breast and colorectal cancer cells regulating the expression of oncogenes [12, 20–26]. Functional validation studies using RNA interference [27, 28] underline their potency as therapeutic targets for cancer [29].

Due to the growth-promoting role in cancer of KDM4s, many KDM4 inhibitors have been constructed for anticancer therapy [30]. These KDM4 inhibitors have been designed as α-KG or 2-OG cofactor mimics, metal cofactor disruptors, histone substrate-competitive inhibitors, or substrate- and cofactor-independent inhibitors. One such example is QC6352, a potent KDM4 inhibitor, that induced antiproliferative effects in, e.g., esophageal squamous cell carcinoma [31] and in breast cancer patient-derived xenograft mice models [32]. JIB-04, a KDM4/5 inhibitor [33], also showed good efficacy in both in vitro and in vivo assessments, in different cancer models [31, 34–38]. GSK-J4, a strong KDM6 inhibitor, also demonstrated inhibition of the enzymatic activity of KDM4 enzymes in vitro with equivalent potency as for KDM6 [39, 40]. However, despite the availability of a broad panel of chemical inhibitors, only a few show promise as potential candidates for clinical translation [30, 41]. Especially for drugs designed to act by binding to the catalytic subunit, specificity is limited as this target site has a high similarity in most lysine demethylase enzymes. This mode of inhibition thus heightens the potential for toxicity.

Despite the convincing functional validation of these proteins as oncogenes using RNA interference [14], the transient effects of siRNA and potential unwanted side effects temper enthusiasm for this technology as a therapeutic approach [42, 43]. Epigenetic editing, encompassing the overwriting of a gene's epigenetic status to modify its expression [44], emerges as a promising avenue [45–50]. Among the available tools, CRISPR-dCas9 (deactivated CRISPR-Cas9) fusion proteins stand out as excellent options for achieving targeted reprogramming of gene expression. The mutations in dCas9 deactivate the nuclease activity of the Cas9 molecule without compromising its genome-selective binding capability when guided by a single-guide RNA (sgRNA) to the specific genomic locus of interest, avoiding the possible off-target genetic mutations associated with gene editing [46, 51, 52]. The fusion of dCas9 with a catalytic domain of an epigenetic enzyme (effector domains (EDs)) enables the"writing"or"erasing"of chromatin marks, such as DNA and histone methylation states, at the target of interest, allowing for modulation of gene expression [53–56]. Since epigenetic modifications can be mitotically stable, the induced modulation of the targeted gene might remain even after removal of dCas9-ED and the sgRNAs [47, 53]. The recently developed CRISPRoff-v2.1 represents a fusion protein comprising DNMT3A/3L and KRAB on the N- and C-terminals of dCas9 [53]. The effector protein KRAB recruits enzymes to enable the methylation of H3K9, whereas DNMT3A-3L deposits a methylation mark to the cytosine nucleotide. Although the simultaneous addition of the repressive histone and DNA methylation modifications ensures sustained repression of gene expression for many genes, even those lacking canonical CpG islands or with a low CpG density [53], it is not maintained for all genes [57].

The direct rewriting of epigenetic signatures to up- or downregulate gene expression has led to numerous successes in preclinical studies [53, 58–60]. The emergence of dedicated epi-editing companies [61] reflects a growing interest in bringing this technology to the clinic, with the initiation of the first clinical trial in 2022 on silencing MYC in hepatocellular carcinoma (NCT05497453) [48]. Here, to overcome the non-selective and low efficacy of KDM4 inhibitors, we investigated the silencing of KDM4-A/B/C by epi-editing using CRISPR-offv2.1 (CRISPRoff). Additionally, we explored the potential synergistic effects of combining epi-editing with epi-drugs to formulate a therapeutic strategy against breast and colon cancer. Notably, simultaneous application of epi-editing with epi-drugs, targeting the same target at both transcript and protein level, respectively, has not been explored to date. Our hypothesis posits that the simultaneous inhibition of gene transcription and protein activity, by epi-editing and epi-drugs, will cooperatively reduce the oncogenic properties of cancer cells.

## Materials and methods

### Plasmid constructs

#### dCas9-effector domains

CRISPRoff (DNMT3A/3L-dCas9-KRAB) from Jonathan S Weissman lab [53] was purchased from Addgene (#167981). Plasmids pMLM3705 (dCas9-VP64) and MLM3636 (sgRNAs) were a kind gift from Keith Joung (Addgene plasmid #47754). We used dCas9-NED (Addgene plasmid #109358) as a negative control [62]. The Super Krüppel associated box (KRAB) domain, SKD, was amplified by PCR (Phusion Hot Start II High-Fidelity DNA polymerase, Thermo Fisher Scientific) and introduced into dCas9-NED to create dCas9-SKD [63]. The plasmids dCas9-G9A, -EZH2, -JARID and -DNMT3A/3L were generated by cloning the corresponding catalytic domains of the respective epigenetic effector proteins into the dCas9-NED backbone vector [62, 64]*.*

#### Single-Guide RNAs (sgRNAs)

All single-guide RNAs were designed using the database (http://crispor.tefor.net/crispor.py) targeting different regions of KDM4A or KDM4B or KDM4C. Six sgRNAs targeting the genomic regions encompassing TSS and/or CpG island were chosen to target KDM4A, similarly five sgRNAs were chosen for KDM4B and four for KDM4C (Supplementary Fig. 1 and see Supplementary Table 1 and Supplementary Table 2). For transient transfections, the sgRNAs were cloned by inserting DNA oligonucleotides containing the 20-bp target region between the two BsmBI sites of MLM3636 (a kind gift from Keith Joung (Addgene plasmid #43860)) for KDM4A and MLM3636-eGFP plasmid for KDM4B and KDM4C [62]. To make stable cells expressing the sgRNA(1)-KDM4A and sgRNA(3)-KDM4A-targeting KDM4A promoter, both sgRNAs were cloned and inserted into the lentiviral sgRNA backbone (the (MS2) zeo backbone from Feng Zhang (Addgene plasmid # 61427)) in the same way as for the MLM3636 plasmids, by inserting DNA oligonucleotides containing the 20-bp target region between the two BsmBI sites of the lentiviral sgRNA plasmid.

### Cell culture

HEK293T, MCF7, HepG2 and C33A, obtained from ATCC, were cultured in DMEM (Gibco, cat. no 41966–052) media supplemented with 10% Fetal Bovine Serum (FBS), 2 mM Glutamine (Lonza, cat. no BE17-605E) and 100 µg/mL of antibiotics (penicillin and streptomycin) (Thermo Scientific cat.no 2441874). HCT116, from ATCC, was maintained in RPMI-1640 (Gibco, cat. no 5200–041) supplemented with 10% Fetal Bovine Serum (FBS), 100 mM sodium pyruvate (Lonza cat.no BE13-115E), 50 mM β-mercapto-ethanol (Merck cat. no 1154330100) and 10 mg/mL gentamicin (Lonza cat.no BE02-012E). The cells were incubated in a static humidified incubator set at 37 °C and 5% CO_2_.

### Transfection

Polyethyleneimine (PEI) was used to perform transient transfections in all cell lines. HEK293T, MCF7, HepG2 and C33A cell lines were plated 24 h before transfection, and HCT116 cells were plated 48 h prior to transfection. The cell lines were seeded in different densities to achieve 70–80% confluence on the day of transfection. The individual sgRNA plasmids targeting KDM4A or KDM4B or KDM4C were pooled to prepare sgRNA(mix)-KDM4A/B/C. The pooled mixtures contained equimolar concentration of individual sgRNA plasmids. For 24-well plate-based transfections, a total of 750 ng of DNA was transfected. The plasmids for dCas9-NED/SKD/VP64/G9A/EZH2/JARID/DNMT3A-3L or CRISPRoff and the sgRNA were mixed in 1:1 ratio in basic DMEM. dCas9-SKD/G9A/EZH2/JARID/DNMT3A-3L were used alone or in combination mixed in 1:1 ratio. The PEI in basic DMEM was added to the DNA-mix to a final ratio of 6:1 (PEI: DNA; w/w). The media were renewed 24 h after transfection.

In experiments followed by sorting, cells were plated in 6-well plates, and 3000 ng of plasmid DNA was transfected. Similar to transfections performed for cells in 24-well plates, the DNA and PEI mixes were prepared in basic DMEM media. The ratio of plasmids encoding the dCas9 variants and sgRNA mixes remained 1:1, and the ratio of PEI to DNA was optimized to 4:1. For transient transfection, MLM3636, without inserted target sequence, was used as negative control (mock), or together with CRISPRoff.

### Cell transduction

Transduction of HCT116 cells was done as described earlier [63]. Briefly, HEK293T cells were transfected with lenti sgRNA1-KDM4A or sgRNA3-KDM4A plasmid (2000 ng), pCMV-ΔR8.91 (1:1 with sgRNA) and pCMV-VSV-G (1:8 with sgRNA) (#8454, Addgene, Watertown, MA, USA) using lipofectamine 3000 transfection reagents (Thermo Fisher) to produce lentiviral particles. The viral supernatant that was harvested after 48 and 72 h was filtered using 0.45 µm pore filter, supplemented with 10 µg/mL polybrene and added to HCT116 cells at 48 h after seeding. The transduced cells were selected on day seven in 2 µg/mL puromycin-supplemented medium for four days and subsequently cultured in 1 µg/mL puromycin-supplemented medium [65].

### Epi-drug preparation

Working stock solution of 10 mM was prepared by dissolving the drug in DMSO (Merck, cat. no 1096780100). The working stock was diluted to final concentrations in 500 µL of culturing media. The compounds EML631, EML951, EML741, UNC0638, EML981 and UNC037 were a kind gift from G. Sbardella’s lab [66–71].

### Epi-editing + epi-drug cell treatment

HEK293T, HCT116, MCF7 and HepG2 cells, 24 h after transfection to express dCas9 (CRISPRoff or dCas9-NED) alone or together with sgRNAs, were treated with QC6252 (1 µM, 0.25 µM or 0.05 µM) or JIB-04 (5 µM) for 24 h before the RNA extraction.

### RNA extraction

Total RNA was extracted using Trizol (Invitrogen, cat no. 21267501). The cell pellets were completely dissolved in Trizol followed by addition of chloroform and subsequent centrifugation at 12000 g for 15 min. The aqueous phase was used for the downstream process. RNA was precipitated by adding an equal volume of isopropanol, followed by incubation at −20 °C for 10 min and centrifugation at 12000 g for 10 min. The RNA pellet was washed twice with 75% ethanol. The RNA pellets were centrifuged at 7500 g for 5 min in between the two ethanol washes. The pellets were resuspended in milli-Q water. The isolated RNA was quantified using nanodrop.

### cDNA Synthesis and RT-qPCR

cDNA was synthesized using the Revertaid Reverse transcriptase kit (Thermo Scientific, cat. no EP0441) and random hexamer primers using 150 ng of RNA isolated from cells at different timepoints. The reaction buffer and synthesis conditions were according to the manual. Further, 15 ng of cDNA was used as template for the RT-qPCR. The template cDNA was supplemented with primers specific for KDM4A/B/C, or GAPDH (Supplementary Table 3) along with FastStart Universal SYBR-green master mix (Roche, cat. no 04913914001). All the reactions were performed in duplicate using ABI ViiA7 real-time PCR system. The data were analyzed based on 2 -∆∆Ct method after normalizing the corresponding samples to Ct values for GAPDH.

### Cell sorting and plating

To perform the fluorescence associated cell sorting (FACS), HCT116 and MCF7 cells were detached, 72 h after transfection, using trypsin (Gibco, cat. no 15400–054). Media were removed after centrifugation at 200 g for 5 min and washed with phosphate-buffered saline (PBS) (Gibco, cat. no 14190–169). The cells were re-suspended in 500 µl of culturing media before sorting. Cells transfected with dCas9-NED or CRISPRoff and the sgRNA plasmids were sorted for m-Cherry and green fluorescent protein (GFP) or blue fluorescent protein (BFP) and GFP, respectively. The sorted cells were collected in culturing media and plated at densities of 2 × 10^4^ cells/well for MCF-7 (96-well plate) and 6 × 10^4^ cells/well for HCT116 (24-well plate) until 21 days from the day of transfection to analyze the change in gene expression of KDM4B and KDM4C. Cells sorted for KDM4A gene expression modulation were collected for genomic DNA extraction.

### Proliferation assay

Effects of modulation of KDM4A expression on cell proliferation were evaluated by Trypan Blue Cell Analysis. After 48 h of transient transfection, the cells were harvested, counted and re-plated in 1:4 ratio in a 24-well plate. Cell count determinations were repeated every day until 96 h after transfection.

### MTS assay

Cell viability was determined using MTS (3-(4,5-dimethylthiazol-2-yl)−5-(3-carboxymethoxyphenyl)−2-(4-sulfophenyl)−2H-tetrazolium) assay. HCT116, MCF7 and HepG2 cells (5 × 10^4^ cells/well) plated in a 24-well plate, were transfected, as described previously, and treated 24 h later with QC6352 at different concentrations (0.05 µM, 0.25 µM and 1 µM for HCT116; 0.25 µM and 1 µM for MCF7; 1 µM for HepG2) for 72 h. MTS solution was added for 3 h at 0.5 mg/mL, and the absorbance was read at a wavelength of 570 nm by CLARIOstar Plus Microplate Reader (BMG labtech).

### Genomic DNA isolation

HCT116 cells were lysed using 500 μl of TE lysis solution (10 mM Tris–HCl pH 8.0; 0.1 mM EDTA pH 8.0; 0.5% SDS; 200 mM NaCl) for 1 h at 37 °C with RNase A (3 μl; 4 mg/ml), followed by incubation for 1 h at 55 °C with Proteinase K (30 μl; 10 mg/ml). DNA was then extracted using an equal volume of chloroform/isoamyl alcohol (24:1). After centrifugation at 12000 g for 5 min, the aqueous phase was collected, and DNA was precipitated at − 80 °C for 3 h by adding 1/5 volume of 1.5 M sodium acetate (pH 5.2), 2.5 volumes of 100% ethanol and 1 μl of 100% glycogen. Samples were centrifuged at 12000 g for 30 min at 4 °C, and the resulting DNA pellets were washed twice with 70% ethanol. DNA was finally resuspended in 10 μl of sterile water and quantified using NanoPhotometer N60 (IMPLEN, DE).

### Pyrosequencing and sample preparation

500 ng of DNA was bisulfite-converted using the EZ DNA methylation Gold Kit (Zymo Research, Irvine, CA, USA) according to the manufacturer’s instructions. For pyrosequencing, bisulfite PCR of a KDM4A region was conducted using bisulfite-specific primers (fw: AGTTATGGGTGATTAGGAGT; rw: CAAAAATAACTACAAACCATACACC; Seq: ATTGGTAGAGGTTTTTAGTGGTAT. Primers were designed using the PyroMark Assay Design 2.0 software (Qiagen whereafter); the PCR product was run on a 1% agarose gel to confirm amplification and predicted amplicon length. The PCR product was sequenced using the Q24 pyrosequencing machine (Qiagen) according to the manufacturer’s instructions. The percentage methylation at each CpG site was determined using the PyroMark Q24 2.0.6.20 software (Qiagen).

### Cell cycle analysis

HCT116 cells (8 × 10^4^ cells/well) were plated in 24-well plate and treated with QC6352 at 1 µM 24 h after CRISPRoff transfection. The cells were collected 48 h after transfection, centrifuged (200 g for 3 min), washed with PBS and suspended in 1 × PBS containing 0.1% sodium citrate, 0.1% NP40 and 50 mg/mL propidium iodide. After 20 min of incubation at room temperature in the dark, cell cycle was evaluated by FACS (NovoCyte Quanteon Flow Cytometer—Agilent).

### Protein histone extraction and Western blot analysis

HCT116 cells were plated in 6-well plate (1 × 10^5^ cells/well) and treated with QC6352 at 0.25 µM 24 h after CRISPRoff transfection. Cells were harvested 48 h and 72 h after transfection (that correspond to 24 h and 48 h after QC6352 treatment) and lysed in 1 ml of Triton Extraction Buffer (TEB) consisting of PBS with 0.5% Triton X-100 (v/v), 2 mM phenylmethylsulfonyl fluoride, and 0.02% (w/v) NaN_3_ for 10 min on ice and centrifuged (450 g at 4 °C for 10 min). The pellet was washed with 500 µl TEB, centrifuged and suspended and incubated in 0.2 N HCl overnight at 4 °C on a rolling table. The supernatant was recovered after centrifugation at 450 g for 10 min at 4 °C and. The protein concentration was determined using a Bradford assay, read by CLARIOstar Plus Microplate Reader (BMG labtech).

Western blot analysis was performed loading 6 μg of protein extracts on 13% polyacrylamide gels. The changes in histone modification were detected by H3K9me3 antibody (Abcam), and histone H4 (Abcam) used as normalization control. Semiquantitative analysis was performed using ImageJ software.

### Data set analysis

The KDM4A expression levels were compared between breast, colon and liver cancer data from TCGA (TCGA-BRCA, TCGA-COAD, TCGA-LIHC) and normal tissue data from GTEX that were obtained using the code in the pipeline [72]. Comparison between colon and normal tissue was also performed for KDM4B and KDM4C. The expression of KDM4s was analyzed in R using a published pipeline. The code from the protocol was adapted to compare KDM4A expression between cancerous and corresponding normal tissues.

### Statistical analysis

Statistical tests were performed using GraphPad Prism 7 software. Comparison between target conditions and controls were conducted using unpaired two-tailed multiple comparison analysis with Tukey's correction. Differences were considered statistically significant at p-value was < 0.05. All data are presented as the mean ± S.D. of three independent, biological replicates, unless stated differently.

## Results

### Modulation of KDM4A expression

To modulate KDM4A gene expression, six sgRNAs targeting the KDM4A promoter (Supplementary Table 1) were strategically designed around the transcription start sites (TSS) as illustrated in Supplementary Fig. 1C. The selected sgRNAs were chosen based on their proximity to the TSS and their minimal predicted off-target effects (Supplementary Table 2). SgRNA(1)-KDM4A and sgRNA(2)-KDM4A are located outside of the CGI and sgRNA(3)-KDM4A to sgRNA(6)-KDM4A target sites in the CGI. SgRNA(3)-KDM4A and sgRNA(5)-KDM4A bind close to the TSS; sgRNA(4)-KDM4A and sgRNA(6)-KDM4A bind more than 208 bps downstream of the TSS. The efficiency of these sgRNAs in modulating KDM4A gene expression was first tested for dCas9-SKD and -VP64 by transient transfection of these effectors and the individual guides in HEK293T and HCT116 cells, without strong effects (Supplementary Fig. 2A, B). Even stable expression of selected sgRNAs-KDM4A (sgRNA(1)-KDM4A and sgRNA(3)-KDM4A) in HEK293T cells did not yield significant improvements upon transient expression of dCas9-SKD or -VP64 compared to dCas9-NED, or to untransfected cells stably expressing only the sgRNAs (Supplementary Fig. 2C). Co-transfection of HEK293T cells to express all guides simultaneously (sgRNA-KDM4A) did also not drastically improve the gene regulatory effect, with dCas9-VP64 leading to a 2.0-fold induction of KDM4A gene expression after 48 h (p = 0.014), and no significant reduction when co-transfected with dCas9-SKD (Supplementary Fig. 2D).

Using the same transfection mixtures in colon (HCT116), breast (MCF7) and cervical (C33A) cancer cells, no robust gene modulation was observed for either dCas9-fusions (with transfection efficiencies of 20–30%) (Supplementary Fig. 2E-G). Only in the hepatocellular carcinoma cells (HepG2), the co-transfection of sgRNA-KDM4A with dCas9-VP64 increased the KDM4A gene expression by 2.2-fold (p = 0.025) compared to cells co-transfected with dCas9-NED (Supplementary Fig. 2H). To improve the degree of downregulation, we tested the sgRNA-KDM4A with various epigenetic dCas9-EDs (effector domains) alone or in combinations in HEK293T cells. Only co-transfection of sgRNA-KDM4A with the combination of dCas9-SKD + dCas9-DNMT3A/3L, or dCas9-EZH2 + dCas9-DNMT3A/3L, resulted in a reduction of KDM4A gene expression compared to dCas9-NED, dCas9-SKD, dCas9-DNMT3A/3L or dCas9-EZH2 transfection alone (Supplementary Fig. 2I).

### Efficient KDM4A downregulation by CRISPRoff induced antiproliferative effect in colon and breast cancer cells 

**Fig. 1:**
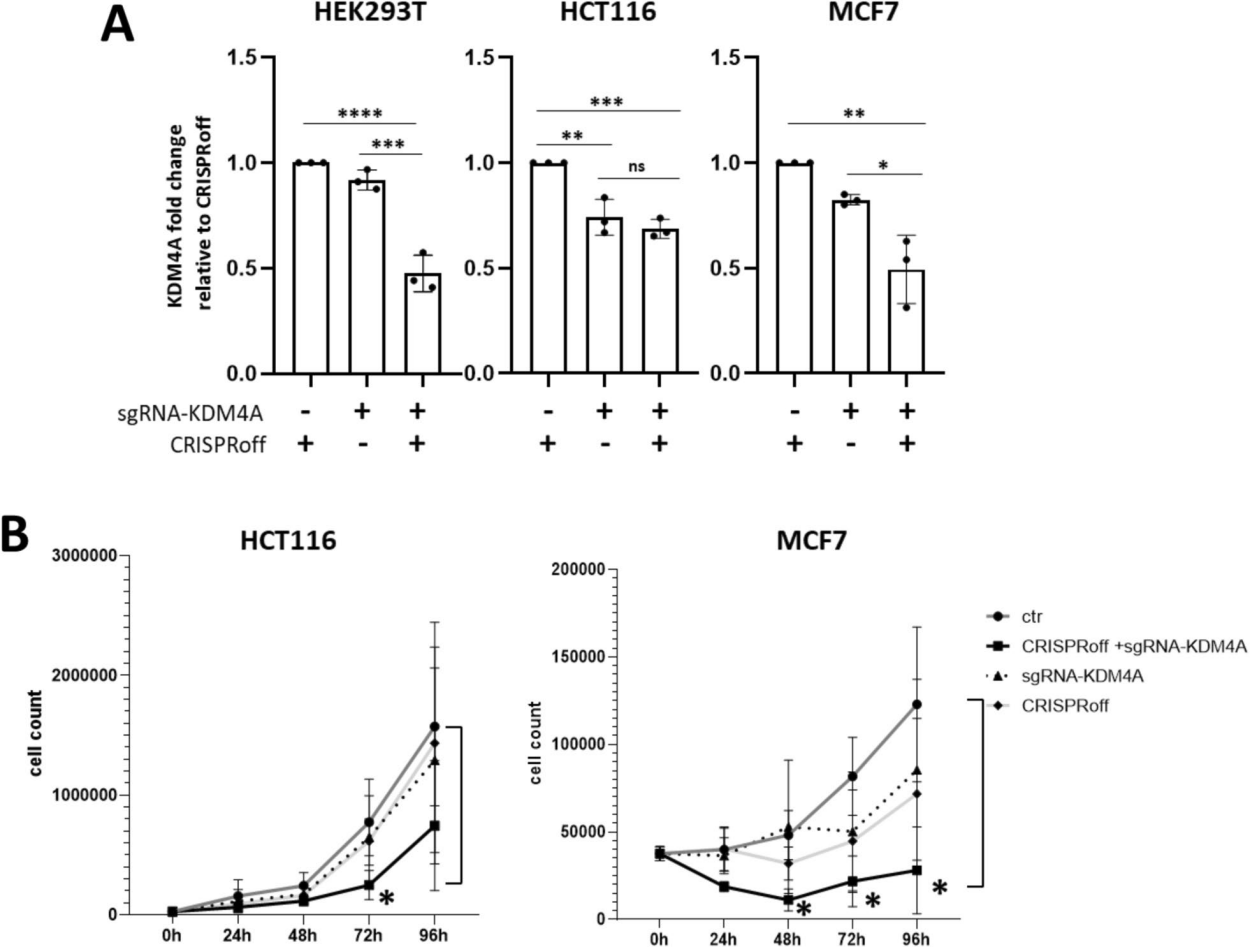
(**A**)KDM4A gene expression reduction by epi-editing using CRISPRoff in HEK293T, HCT116 and MCF7; (**B**) Proliferation assay in HCT116 and MCF7 cells upon downregulating KDM4A. The dark gray lines show the untransfected cells (symbol: circle), in solid black lines the cells transfected with CRISPRoff and sgRNA-KDM4A (symbol: square), the dashed lines show the cells transfected only with sgRNA-KDM4A (symbol: triangle), and in light gray the cells only with CRISPRoff (symbol: diamond)

Based on the improved effects of the combination of epigenetic effectors, HEK293T cells were transiently transfected to express CRISPRoff [53], wherein one dCas9 protein is fused to DNMT3A/L and KRAB. The effects of CRISPRoff with sgRNA-KDM4A on KDM4A gene expression were compared to only CRISPRoff or only sgRNA-KDM4A as controls. In HEK293T cells, 2 days post-transfection, KDM4A expression was reduced to 47% (p < 0.0001) when compared to cells transfected to express only CRISPRoff and to 52% (p < 0.001) compared to cells transfected to express only sgRNA-KDM4A (Fig. 1A).

Following this validation of the sgRNA-KDM4A with CRISPRoff to downregulate KDM4A in HEK293T cells, the efficacy was explored in HCT116 colon and MCF7 breast cancer cells. Two days post-transfection, both HCT116 and MCF7 cells expressing CRISPRoff along with the targeting sgRNA-KDM4A exhibited lower expression of KDM4A, compared to the controls. In HCT116 cells, the expression of KDM4A was reduced to 69% (p < 0.001) compared to CRISPRoff control, although the induced repression was similar for the cells expressing only targeting sgRNA-KDM4A (p = 0.42). In MCF7 cells, KDM4A gene expression was reduced to 50% (p = 0.001) compared to CRISPRoff control and reduced to 59% compared to sgRNAs only (p = 0.012) (Fig. 1A).

In HCT116 cells, DNA methylation analysis following co-transfection with CRISPRoff and sgRNA-KDM4A demonstrated an increase in methylation at the KDM4A promoter region, compared to cells transfected with either CRISPRoff or sgRNA-KDM4A alone (Supplementary Fig. 3A).

Putative off-target binding was assessed for the sgRNAs allowing up to three mismatches (Supplementary Table 2). No off-target effects were observed following transfection with CRISPRoff and sgRNA-KDM4A, although sgRNA(5)-KDM4A was localized within 1 kb of the LINC00959 transcription start site (Supplementary Fig. 3B).

The functional effects on cell proliferation induced by the KDM4A downregulation were evaluated up to 96 h post-transfection. Unlike HEK293T cells, where the downregulation of KDM4A did not impact cell growth (Supplementary Fig. 3C), both HCT116 and MCF7 cancer cells exhibited reduced cell proliferation following the decrease in KDM4A gene expression induced by CRISPRoff plus sgRNA-KDM4A compared to the controls (Fig. 1B). This confirms the oncogenic role of KDM4A in colon and breast cancer. Taken together, these data suggest that CRISPRoff can efficiently reduce KDM4A gene expression, thereby promoting an anticancer effect.

### Inhibition of KDM4 protein activity increased its gene expression

**Fig. 2:**
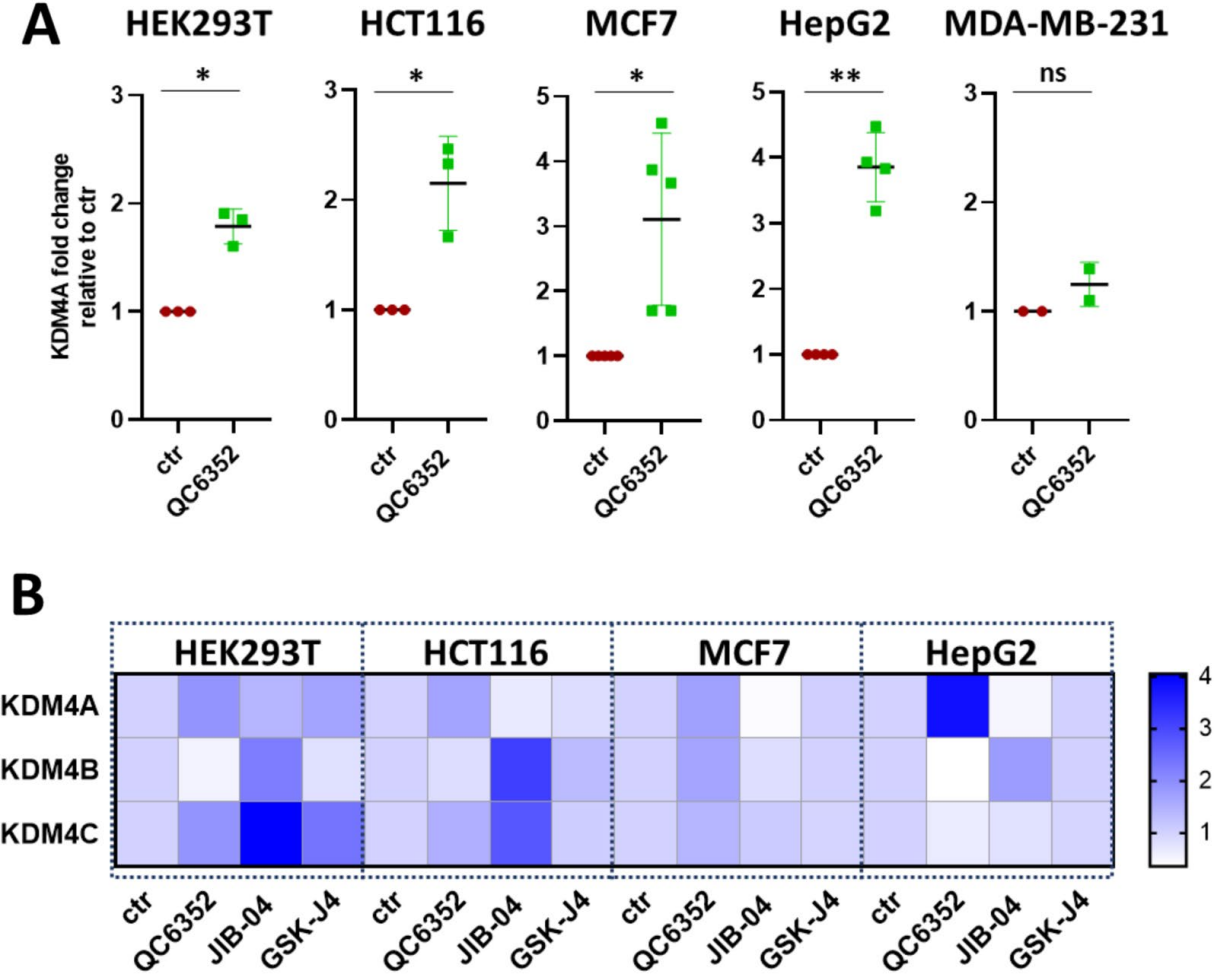
(**A**) KDM4A expression evaluation in HEK239T, HCT116, MCF7, HepG2 and MDA-MB-231 cells treated with KDM4 inhibitor, QC6352, for 24 h at 5 µM, or with DMSO (ctr); (**B**) KDM4A/B/C expression in HCT116, MCF7, HEK239T and HepG2 cells after 24 h of treatment with the pan-KDM inhibitors JIB-04 and KDM6 inhibitor GSK-J4 at 10 µM (data represent the mean of three independent experiments)

To repress KDM4A protein activity, cancer cells were treated with small-molecule KDM4 inhibitors. Interestingly, 24 h after QC6352 treatment, we observed a > 1.8-fold increase in KDM4A RNA expression across different cell lines (HEK293T 1.8-fold, p = 0.014; HCT116 2.2-fold, p = 0.043; MCF7 3.1-fold, p = 0.024; HepG2 3.9-fold, p = 0.002) (Fig. 2A). For MDA-MB-231 breast cancer cells, no such increase in expression of the gene encoding the targeted protein was detected. Upregulation of KDM4A expression, although less pronounced, was also identified in HEK293T cells for GSK-J4 (1.67-fold, p = 0.026). No significant regulation of KDM4A was observed in either cell lines following treatment with the pan-KDM inhibitor JIB-04 (Fig. 2B).

Conversely, JIB-04 did induce the RNA expression of KDM4B and −4C (2.2- and 4.1-fold increase in HEK293T (p = 0.043, p = 0.035) cells and a 3.2- and 3.1-fold increase in HCT116 (p < 0.0001; p = 0.03) (Fig. 2B). QC6352 had a more moderate effect: QC6352 induced a 1.9-, 1.6- and 1.7-fold increase in KDM4C gene expression in HEK293T, HCT116 and MCF7 cells, respectively. Additionally, there was a 1.7-fold increase in MCF7 for KDM4B (p = 0.015). For HepG2, no significant compensatory upregulation regulation was observed for KDM4B and KDM4C for any of the KDM4 inhibitors. No induction was also detected in any of the cell lines following treatment with GSK-J4 (Fig. 2B).

Next, 12 epi-drugs targeting other classes of epigenetic players were tested to evaluate whether the observed effect was related to a specific epigenetic mechanism. In HEK293T cells, treatment with the other epi-drugs did not induce KDM4A gene upregulation compared to the control (Supplementary Fig. 3D). Also, neither epi-compound induced the expression of KDM4A in HCT116, MCF7, or MDA-MB-231 cells. However, notably, an upregulation of KDM4A expression was detected in HepG2 cells following treatment with SAHA, MS275 and UVI5008 (Supplementary Fig. 3D). Altogether, these results suggest that KDM4 inhibitors can increase the expression of KDM4A, B and C in a cell context-dependent manner.

### Epi-editing prevented the epi-drug-induced KDM4A gene upregulation

**Fig. 3:**
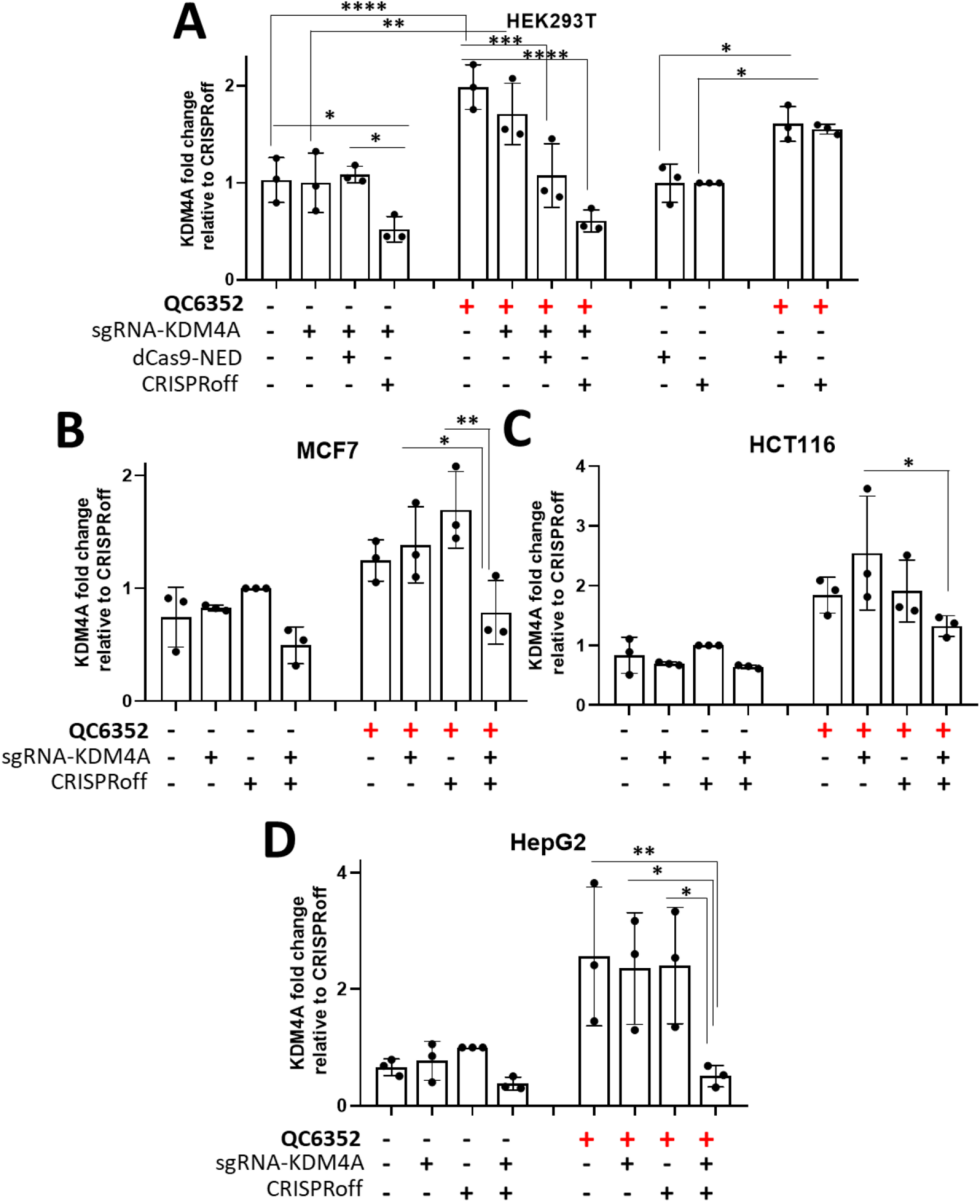
KDM4A expression after QC6352 (1 µM) treatment and/or epi-editing using CRISPRoff + sgRNA-KDM4A in (**A**) HEK293T, (**B**) MCF7, (**C**) HCT116 and (**D**) HepG2 cells. All samples were normalized to cells transfected only with CRISPRoff. The first samples of each graph show the negative control (mock): cells transfected with MLM3636, without inserted target sequence

We hypothesized that the compensatory gene upregulation induced by epi-drugs could be mitigated through epi-editing. Again, in HEK293T cells, transiently transfected to express sgRNA-KDM4A along with CRISPRoff, KDM4A expression was reduced to 50% compared to cells transfected with empty MLM3636 (mock transfected) (p = 0.048) and compared to cells transfected with sgRNA-KDM4A + dCas9-NED (dCas9 protein without fused EDs) (p = 0.023)(Fig. 3A, left panel). When the cells were treated for 24 h with QC6352, a 1.5- to twofold induction of KDM4A expression was again observed compared to control cells (mock (p < 0.0001) and only sgRNA-KDM4A (p = 0.003); Fig. 3A, middle panel and dCas9-NED (p = 0.012) and CRISPRoff without sgRNA-KDM4A (p = 0.026); Fig. 3A, right panel). Intriguingly, the presence of dCas9-NED (dCas9 protein + sgRNAs) on the KDM4A promoter region was enough to prevent the upregulation induced by QC6352 (p = 0.0002) (Fig. 3A, middle panel), while the expression of CRISPRoff and sgRNA-KDM4A even further reduced the expression of KDM4A to 30% compared to QC6352 treated cells (p < 0.0001).

We then investigated the impact of epi-editing on preventing the compensatory gene upregulation in other cell types. HCT116, MCF7 and HepG2 cells were treated with 1 µM QC6352 or vehicle, 24 h after sgRNA-KDM4A + CRISPRoff transfection. In MCF7 and HCT116 cancer cells, the CRISPRoff + sgRNA-KDM4A complex completely blocked KDM4A gene upregulation induced by QC6352, although no further repression was achieved (Fig. 3B, C). Relative to cells transfected to only express sgRNA-KDM4A or only CRISPRoff, treatment with QC6352 failed to upregulate KDM4A gene expression in MCF7 cells when transfected to express CRISPRoff + sgRNA-KDM4A. KDM4A expression was actually lowered to 45% (p = 0.033) (p = 0.0012), compared to sgRNA-KDM4A or only CRISPRoff, respectively (Fig. 3B). In HCT116 colon cancer cells, CRISPRoff + sgRNA-KDM4A significantly reduced the QC6352 effect compared to cells transfected only with sgRNA-KDM4A (40% left; p = 0.013) (Fig. 3C). In HepG2 cells, the > 2-fold induction of KDM4A expression was completely prevented, with 89% of gene downregulation (p = 0.008) compared to the treated mock-transfected cells (Fig. 3D). These data point to a potential synergistic effect of co-treating cancer cells with epi-editing and epi-drugs.

### The combination epi-editing plus epi-drug improved the anticancer effects

**Fig. 4:**
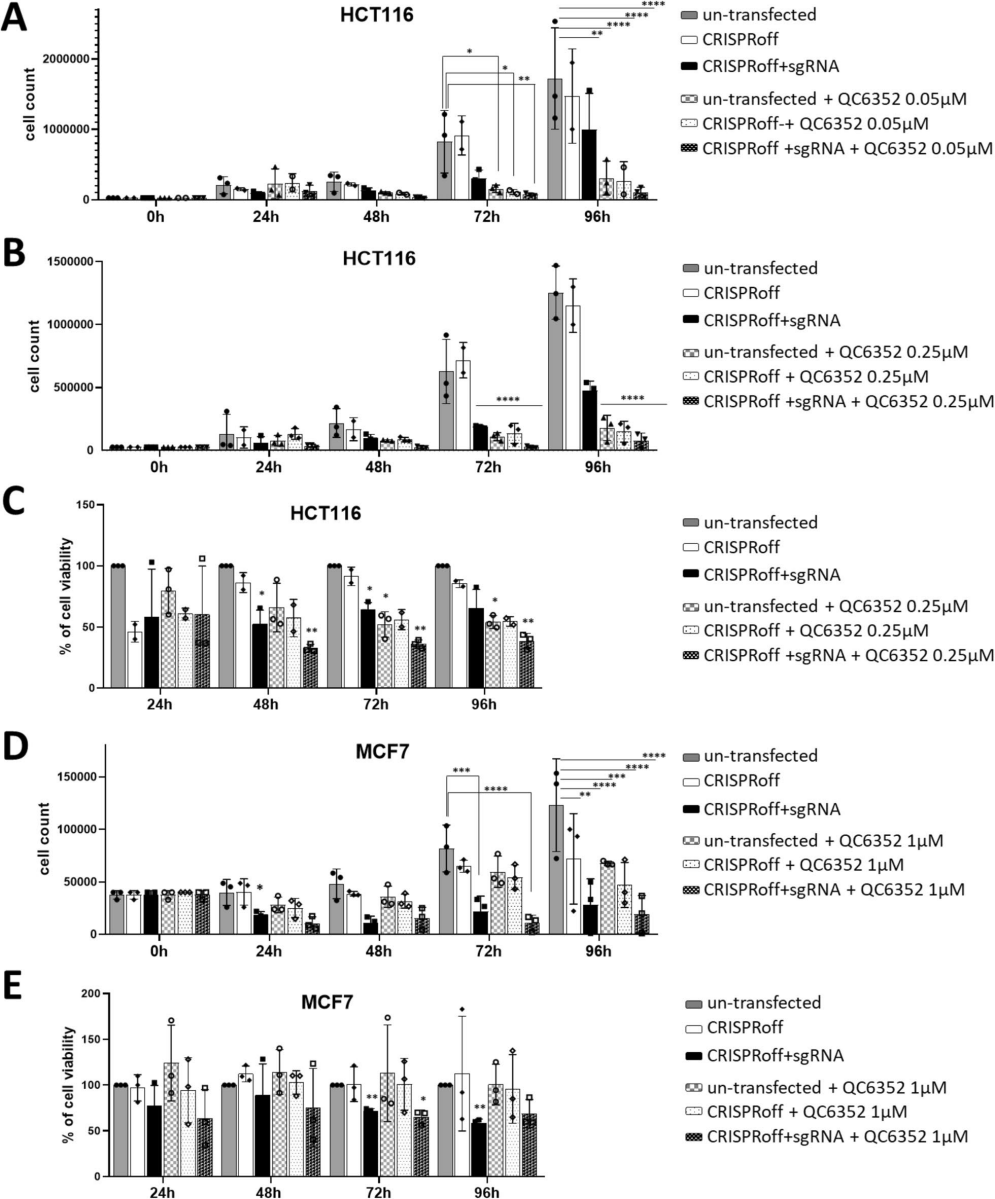
Proliferation assay in (**A-B**) HCT116 and (**D**) MCF7 cells with/without sgRNA-KDM4A (sgRNA) treated with QC6352 at (**A**) 0.05 µM, **(B)** 0.25 µM and (**D**) 1 µM or DMSO (ctr); MTS assay in (**C**) HCT116 and (**E**) MCF7 cells with/without sgRNA-KDM4A treated with QC6352 at (**C**) 0.25 µM and (**E**) 1 µM or DMSO (ctr). The dark gray bars show the untransfected cells (symbol: circle), in white bars the cells only with CRISPRoff (symbol: diamond), in black the cells transfected with CRISPRoff + sgRNA-KDM4A (symbol: square). The solid bars represent the cells only transfected with epi-editing, and the dashed bars, the cells treated with QC6352 after transfection

We next assessed the potential improvement in anticancer effects through the combination of gene repression and inhibition of protein activity in cancer models. HCT116 colon cancer cells were transiently transfected to express CRISPRoff + sgRNA-KDM4A, followed by a 24 h treatment with QC6352 at 0.05 and 0.25 µM. This combined approach resulted in a significant inhibition of proliferation for up to 96 h, improving the effects observed for cells treated solely with QC6352 at both concentrations, or with CRISPRoff + sgRNA-KDM4A alone (Fig. 4A-C). At 72 and 96 h, QC6352 and CRISPRoff + sgRNA-KDM4A, alone or in combination, exhibited a pronounced reduction of HCT116 cell proliferation compared to the control cells (p < 0.0001). Moreover, the combination of QC6352 and CRISPRoff + sgRNA-KDM4A, while not eliciting a significant enhancement in proliferative inhibition compared to QC6352 alone, markedly attenuated cell proliferation at 96 h when compared to cells expressing CRISPRoff + sgRNA-KDM4A alone (< 0.0001) (Fig. 4A, B). The combination of epi-drug and epi-editing, when assessed through MTS assay, induced a significant block in growth as early as 48 h compared to control cells (p = 0.001; 72 h: p = 0.003; 96 h: p = 0.012). However, only at 72 h the combination of QC6352 and CRISPRoff + sgRNA-KDM4A reduced cell proliferation more effectively relative to cells expressing CRISPRoff + sgRNA-KDM4A alone (p = 0.027) (Fig. 4C).

The experiment was also evaluated at 1 µM of QC6352 (Supplementary Fig. 4A-B) which already alone induced strong antiproliferative effects, making it difficult to distinguish any additional impact from the epi-editing + drug combination when assessed by standard proliferation assays (Supplementary Fig. 4A). However, using the MTS assay, the combination of QC6352 with CRISPRoff + sgRNA-KDM4A induced a block in proliferation from 48 up to 96 h. This effect was stronger than that seen with CRISPRoff + sgRNA-KDM4A alone and showed enhanced anticancer activity compared to QC6352 monotherapy (Supplementary Fig. 4B).

In MCF7 breast cancer cells, the downregulation of KDM4A by epi-editing alone induced a substantial inhibition in proliferation at 72 h and 96 h compared to control cells and to cells transfected with only CRISPRoff (Fig. 4D, E). Under these experimental conditions, epi-editing even outperformed the epi-drug approach. Also, the combination epi-editing with QC6352 did not further improve the anticancer effect when tested at 1 µM (Fig. 4D, E) and 0.25 µM (Supplementary Fig. 4C).

Unexpectedly, neither CRISPRoff + sgRNA-KDM4A, the inhibitor QC6352, nor their combination resulted in any anticancer effects in HepG2 cells (Supplementary Fig. 4D). Similar results were obtained when KDM4A was downregulated to 40–60% by siRNA (Supplementary Fig. 4E, F).

For HCT116 cells, we also assessed cell cycle phases, but no differences were observed after transfection with CRISPRoff + sgRNA-KDM4A alone or in combination with QC6352 (Supplementary Fig. 5A). Interestingly, only the combination epi-editing + epi-drug significantly inhibited KDM4A functioning, as demonstrated by an increase in H3K9me3 histone marks (Supplementary Fig. 5B). All these results support the hypothesis that the epigenetic editing approach can improve epi-drug anticancer effects for selected cell types by targeting the same protein both at its RNA and at the protein level.

### CRISPRoff downregulated KDM4B and KDM4C expression in cancer cells

**Fig. 5:**
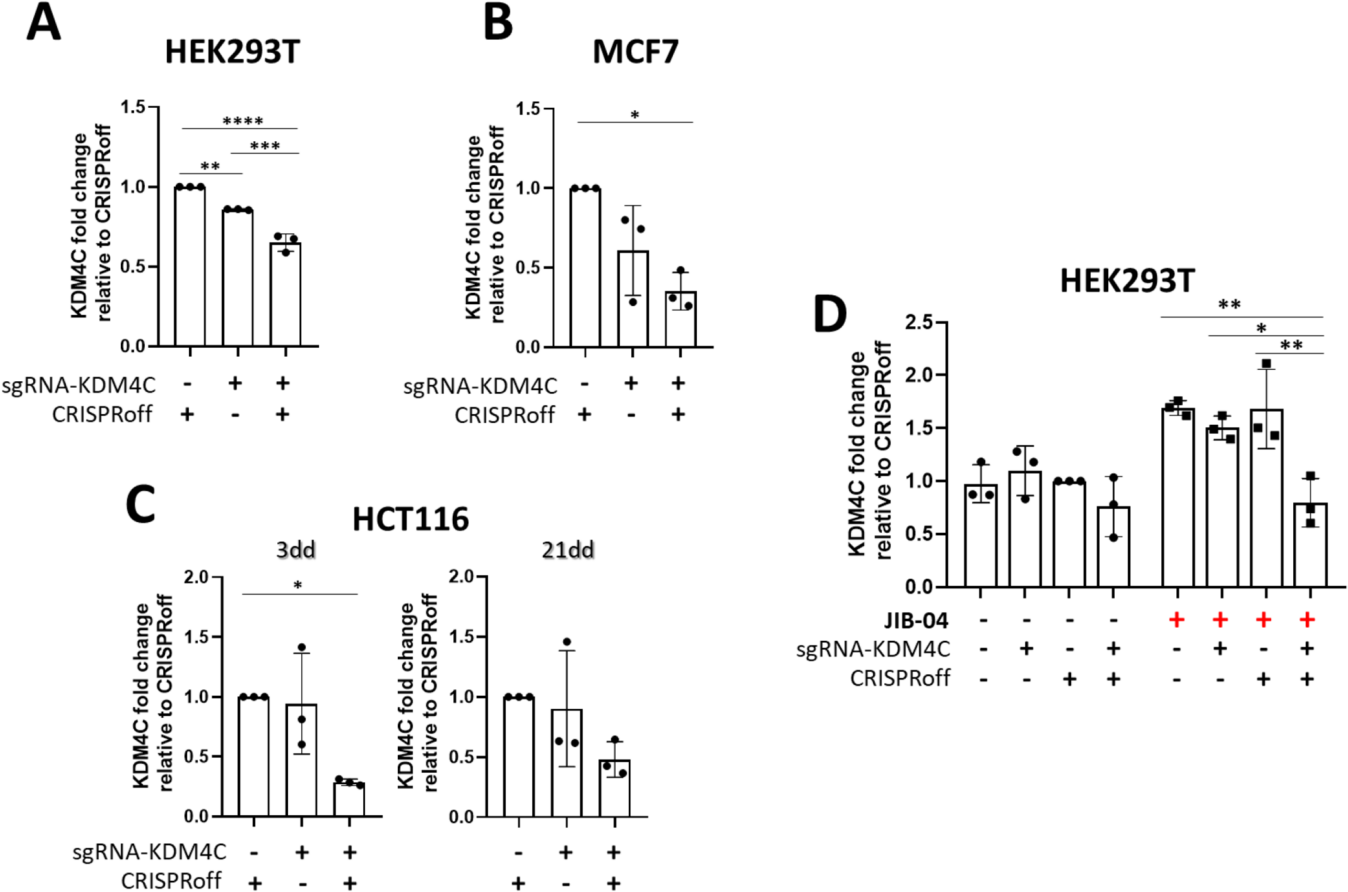
KDM4C gene expression reduction by epi-editing in (**A**) HEK293T and (**B**) sorted MCF7 cells 3 days after CRISPRoff and sgRNA-KDM4C transfection; (**C**) KDM4C gene evaluation in sorted HCT116 cells after 3 and 21 days after transfection; (**D**) KDM4C expression in HEK293T cells after JIB-04 (5 µM) treatment and CRISPRoff transfection

As for KDM4A, sgRNAs were also designed to target KDM4-B and -C around the transcription start sites (TSS)(Supplementary Fig. 1D-F and Supplementary Table 1). Although no substantial modulation was achieved for VP64 and SKD (Supplementary Fig. 6 A, B), downregulation through epi-editing by CRISPRoff was successful for KDM4C upon transient transfections in HEK293T cells (Fig. 5A). 2 days post-transfection KDM4C expression was reduced to 65% (p < 0.0001) compared to control cells expressing only CRISPRoff and 24% (p < 0.001) compared to those expressing only sgRNA-KDM4C (Fig. 5A).

To increase detection sensitivity, MCF7 and HCT116 cells were sorted for BFP and GFP expression, 3 days post-transfection. The sorted cells expressing CRISPRoff along with the targeting sgRNA exhibited lower expression of KDM4C (Fig. 5B, C) compared to their controls (35% left (p = 0.01) and 28% (p = 0.027) of gene expression left in MCF7 and HCT116 cells, respectively). Sorted HCT116 cells were subsequently sub-cultured to assess maintenance of the effect. The repression to 28% at day 3 was maintained to 48% downregulation of KDM4C until 21 days post-transfection (Fig. 5C).

The gene downregulation of KDM4B was less pronounced compared to KDM4C. In HEK293T cells, KDM4B expression was only reduced to 80% compared to the control cells (Supplementary Fig. 6C). In MCF7 cells, the expression of KDM4B was reduced to 69% (Supplementary Fig. 6D), and in HCT116 cells, it reached 65% left at 3 days (p = 0.014) which was maintained up to 21 days after transfection (Supplementary Fig. 6E), compared to control (CRISPRoff). In both cancer cell lines, the reduction in KDM4B expression was similar in the cells co-transfected with targeting sgRNAs, with or without CRISPRoff. Taken together, these data indicate that CRISPRoff effectively reduces the expression of KDM4C in various cell lines and this downregulation was maintained in HCT116 colon cancer cells.

### CRISPRoff prevented the KDM4C gene upregulation induced by JIB-04 treatment

We hypothesized that, similar to KDM4A, epi-editing might prevent the upregulation of KDM4C gene expression induced upon KDM4 protein inhibition by JIB-04 (Fig. 2B). Therefore, we transfected HEK293T cells to express CRISPRoff + sgRNA-KDM4C to reduce KDM4C expression to 47% compared to mock (Fig. 5D). A 1.7- to 1.5-fold induction of KDM4C expression was observed in all control cells (mock, only sgRNA-KDM4C and CRISPRoff without sgRNA-KDM4C) treated with JIB-04 at 5 µM. CRISPRoff + sgRNA-KDM4C not only prevented this JIB-04 induced upregulation, but importantly, further reduced KDM4C expression compared to all the control cells (versus mock: 47% left (p = 0.003), versus only sgRNA-KDM4C: 53% left (p = 0.030), and versus CRISPRoff without sgRNA-KDM4C: 47% left (p = 0.004), Fig. 5D), to the extend similar as observed in the absence of the drug. Thus, these data confirmed that, as for KDM4A, epigenetic editing can prevent the compensatory KDM4C gene upregulation induced by KDM4 inhibitor treatment.

## Discussion

In this study, we describe an intriguing combinatorial approach of simultaneous gene-protein targeting using epi-editing together with epi-drug strategies. We choose to target members of the histone-lysine demethylase KDM4 family as models, as these are seen as promising therapeutic targets and KDM4A-targeting epi-drugs are in development, mainly against cancer. However, so far, no clinical trial evaluating the efficacy of KDM4 inhibitors has been reported due to inefficiency and lack of specificity. We here address both aspects as we demonstrate i) epi-drug-induced compensatory target gene expression upregulation likely affecting efficacy and indicating a previously unnoticed potential resistance mechanism for epi-drugs and ii) a gene-specific approach to silence the epi-enzyme in a gene-targeted manner (epigenetic editing) acts synergistically as it can prevent the compensatory upregulation. Since gene upregulation is a key resistance mechanism in conventional chemotherapy, our dual targeting platform has the potential to enhance not only the efficacy of epi-drugs but also that of chemotherapeutics and other treatments.

Using the chemical KDM4 protein inhibitor QC6352 as well as KDM4A silencing by epi-editing, we corroborated the ability of inhibiting KDM4 to reduce cancer cell viability [31, 32, 73] for breast and colorectal cancers [12, 74]. Indeed, we confirmed overexpression of KDM4A in theses tumor types, while no significant overexpression of KDM4A was observed in patient hepatocellular carcinoma tissue compared to normal tissue in our TCGA dataset analyses. Conversely, another group has reported upregulation of KDM4A in hepatocellular carcinoma and demonstrated a functional oncogenic role for KDM4A, as its overexpression promoted cell proliferation [75, 76]. Our studies, however, do not demonstrate any involvement of KDM4A in HCT116 hepatocellular carcinoma proliferation, neither through epi-editing nor by siRNA downregulation. These controversial data might indicate that only in certain subtypes and/or under specific circumstances hepatocellular carcinoma cells can benefit from targeting KDM4A.

We also observed cell-type dependency in the upregulation of KDM4 RNA expression upon KDM4 epi-drug treatment. Although the cause of overexpression of KDM4 genes in cells treated with QC6352 and with JIB-04, a pan-KDM4 inhibitor, needs further mechanistic assessment, this event may represent one of the mechanisms underlying intrinsic as well as potential acquired resistance of such epi-drugs. Since such overexpression of the target gene weakens the potency of the epi-drug, requiring higher dosages, prevention of this compensatory effect will improve efficacy while aggravating dose-limiting toxicities. We are the first to explicitly address this mechanism for epi-drugs, which only been noted before for a BET inhibitor [11]. Further investigations are needed to assess the frequency of this phenomenon for other epi-drugs. The upregulation of KDM4A after treatment with QC6352 is observed in all tested cell models, except for MDA-MB-231 triple-negative breast cancer cells, which is the only tested cell model mutated for p53. It thus is tempting to hypothesize that the expression of p53 may play a role in the upregulation of KDM4A following QC6352 treatment. However, the increase in p53 upon DNA damage induced a decrease in KDM4A levels [77]. The involvement of p53 in inducing the compensatory upregulation is not straightforward and likely can only partially explain the gene regulation induced by QC6352. This will be part of further investigations, by assessing double knockout cell lines for p53, such as HCT116 p53 -/-.

To prevent/reduce drug-induced upregulation of gene expression, as well as other challenges (specificity, toxicity) faced by epi-drugs [78, 79], we propose epigenetic editing as an attractive method to repress the expression of the target gene [46–49, 56]. In contrast to the efficient repression by CRISPRoff, the KRAB repressive domain, as used in dCas9-SKD, induced gene downregulation only for KDM4A and only in HEK293T cells. A more robust reduction in KDM4A gene expression was achieved through a combination of DNMT3A/3L with SKD or EZH2 (writing H3K27me3) in HEK293T cells, confirming previous studies assessing combination of epigenetic effectors [47, 53–55, 80]. These studies also demonstrate the maintenance of the induced effects, albeit not for all gene targets [54, 57, 81]. Using CRISPRoff, we also achieved repression up to 21 days for KDM4C, but not for KDM4A. The permissivity of a gene for long-term epigenetic downregulation upon a single administration of epigenetic editors is an important research focus as it paves the way for highly effective epigenetic anticancer therapies. We also found that not only CRISPRoff, but even the dCas9-sgRNA complex, is capable of suppressing drug-induced upregulation of the KDM4A gene across all tested cell lines. These findings suggest that the physical presence of the dCas9-sgRNA complex at the target gene promoter is sufficient to (transiently) prevent the drug-induced upregulation. While further investigation is warranted to elucidate the precise mechanisms involved, this observation highlights a potential steric hindrance-mediated repression exerted by dCas9-sgRNA binding.

Ultimately, the achievement of sustained gene silencing via epi-editing has demonstrated an efficacy comparable to gene editing [58, 59], but with the added advantages of being reversible and leaving the genome undamaged. Compared to gene editing using CRISPR-Cas, CRISPR-dCas9 overcomes ethical concerns as it mitigates the risk of unintended mutations through the loss of DNA cleavage activity. The direct rewriting of epigenetic signatures by epigenetic editing has led to numerous successes in preclinical cancer models and several companies are working to bring epi-editing into the clinic [48, 61]. In addition to the epi-editor which has already entered clinical trials to silence MYC in hepatocarcinoma ((OTX-2002; NCT05497453) [48], several others are in the preclinical development phase, showing promising results not only for anticancer therapies but also for, e.g., neurological treatments (CLAIRIgene Company SLS-004 and CLRI-005). However, there are still numerous challenges to overcome, including efficient and specific delivery. Although numerous advancements have been made to reduce off-target and undesired immunological effects [82–84], to date, the most effective and widely used formulation appears to be viral delivery (AAV) [85, 86]. However, numerous cases have been reported in which viral administration led to a significant and sometimes lethal autoimmune reaction [87]. In this regard, lipid nanoparticles (LNPs), also employed in the clinical epi-editing trial, have demonstrated promising activity in recent years without showing toxic effects [88, 89]. Altogether, reprogramming RNA expression by epigenetic editing allows for inhibition of proteins that show (compensatory) upregulation but are difficult to target by conventional (chemo)therapy.

Dual targeting of a protein by an epi-drug together with reducing the RNA expression of the same target to achieve synergy (or, for example, of a related protein causing drug resistance), could be applied to many other drug targets. Inspired by the initiation of the first epi-editing clinical trial for hepatocarcinoma, we show here that a combinatorial approach of epi-editing and epigenetic drugs holds promise as a therapeutic strategy against cancer.

## Conclusions

With this study, we are the first to demonstrate that epi-drug administration can upregulate the gene expression of the drug’s target gene. Additionally, we discovered that by combining epigenetic editing with epi-drug treatment, we can counteract this upregulation and achieve effective downregulation of the targeted genes. This combined approach represents a promising strategy for cancer treatment.

However, despite the clear advantage of using a synergistic approach to strengthen the antitumor response, further investigations are warranted to ascertain the broad applicability of this approach across diverse target types. Moreover, unlike the administration of epi-drugs, which faces fewer challenges, epi-editing requires further studies and explorations to improve cellular availability and safety [90, 91].

## Supplementary Information

Additional file1

## Data Availability

All data generated or analyzed during this study are included in this article.
