# Supplementary material for: Epigenetic editing and epi-drugs: a combination strategy to simultaneously target KDM4 as a novel anticancer approach
Source: Clin Epigenetics. 2025 Jun 19;17:105. doi: 10.1186/s13148-025-01913-0 (PMC12177974; doi:10.1186/s13148-025-01913-0)
Supplement: Supplementary file 1 — Additional file1 [file 13148_2025_1913_MOESM1_ESM.docx]

**Epigenetic editing and epi-drugs combination strategy to simultaneously target KDM4 as a novel anti-cancer approach**

Federica Sarno ^1,2^, Jim J. Jacob^1^, Roos E. Eilers^1^, Angela Nebbioso ^2,4^ Lucia Altucci ^2,3,4^, Marianne G. Rots^1^

**Human (GRCh38/hg38)**

| # | **Sequences** | **Target** | **Chr.** | **strand** | **start** | **end** | **TSS** | **CpG island** |
| --- | --- | --- | --- | --- | --- | --- | --- | --- |
| **SgRNA(1)-KDM4A** | **AGTGGGCCGGTCCGGTAGAT** | KDM4A | 1 | - | 43649855 | 43649874 | -288 | OUT |
| **SgRNA(2)-KDM4A** | **CTCTAGCTGGATCGCGAAGC** | KDM4A | 1 | - | 43649942 | 43649961 | -201 | OUT |
| **SgRNA(3)-KDM4A** | **GAGTTTCGGCCTTCGCCTGC** | KDM4A | 1 | + | 43650159 | 43650178 | +16 | IN |
| **SgRNA(4)-KDM4A** | **GCGGCCGAGCTTCACTCGCCA** | KDM4A | 1 | + | 43650493 | 43650513 | +351 | IN |
| **SgRNA(5)-KDM4A** | **GATCGGCCAGTGGCGACAGC** | KDM4A | 1 | + | 43650193 | 43650212 | +51 | IN |
| **SgRNA(6)-KDM4A** | **ACATGCCGATCACTCACCGC** | KDM4A | 1 | - | 43650351 | 43650370 | +208 | IN |
| **SgRNA(1)-KDM4B** | **GCGGTTGGGGCGTTAACCGCC** | KDM4B | 19 | + | 4969395 | 4969415 | +279 | IN |
| **SgRNA(2)-KDM4B** | **GCGCTCGCGGGGGGGCTTATA** | KDM4B | 19 | - | 4969086 | 4969106 | -30 | IN |
| **SgRNA(3)-KDM4B** | **GAGGGACCCTAACTATTCCGA** | KDM4B | 19 | + | 4968369 | 4968389 | -747 | OUT |
| **SgRNA(4)-KDM4B** | **CGCCCGACGACCGCACAGAG** | KDM4B | 19 | - | 4968745 | 4968764 | -371 | IN |
| **SgRNA(5)-KDM4B** | **CGACATTAAGGGGTGCCGGC** | KDM4B | 19 | - | 5131266 | 5131285 | +104 | OUT |
| **SgRNA(1)-KDM4C** | **GAGGCCGCCATAGGTGCGCGT** | KDM4C | 9 | + | 6757635 | 6757655 | -348 | IN |
| **SgRNA(2)-KDM4C** | **GCGGGTACCGAACCTCGGTAA** | KDM4C | 9 | - | 6758435 | 6758455 | +452 | IN |
| **SgRNA(3)-KDM4C** | **GAGAAACGAGTTCAAGCGCG** | KDM4C | 9 | + | 6756848 | 6756867 | -1135 | OUT |
| **SgRNA(4)-KDM4C** | **CCGCCGTCTACACCGCATCA** | KDM4C | 9 | - | 6758780 | 6758799 | +978 | IN |

**Supplementary Table 1**. sgRNAs specific for KDM4A, KDM4B and KDM4C: sequences and chromosome position.

| **SgRNA_**  **KDM4A** | **MIT**  **Specificity score (for KDM4A)** | **CFD**  **Specificity score (for KDM4A)** | **Off target gene** | **Mismatches** | **Chromosome position** | | **Distance from TSS** | **MIT**  **Off-score** | **CFD**  **Off-score** |
| --- | --- | --- | --- | --- | --- | --- | --- | --- | --- |
| **SgRNA_2** | 98 | 99 | RP11-334A14.8-SLC1A7 | 3 | chr1:53124489-53124511 | intergenic | 18.107 | 0.02 | 0.047 |
| **SgRNA_3** | 93 | 96 | Clostridiales-1-RP11-1094H24.4 | 3 | chr17:50046685-50046707 | intergenic | 9,389 | 0.11 | 0.00 |
| **SgRNA_4** | 96 | 97 | EGF-ELOVL6 | 3 | chr4:110032467-110032489 | intergenic | 166,018 | 1.01 | 0.41 |
|  |  |  | PACRG-RP1-257A15.1 | 3 | chr6:162896290-162896312 | intergenic | 168,278 | 0.08 | 0.17 |
|  |  |  | PLCB1-PLCB1-IT1 | 3 | chr20:8205360-8205382 | intergenic | 73,082 | 0.31 | 0.018 |
| **SgRNA_5** | 88 | 95 | AC021224.1-GAREM | 2 | chr18:32422907-32422929 | intergenic | 47,944 | 0.75 | 0.18 |
|  |  |  | RP11-18B16.2-AL157702.1 | 2 | chr9:113690185-113690207 | intergenic | 39,449 | 0.74 | 0.079 |
|  |  |  | RN7SL308P-ZNF704 | 3 | chr8:80785624-80785646 | intergenic | 89,078 | 0.31 | 0.02 |
|  |  |  | XX-FYM637E10_5.1-PPP2R2D | 2 | chr10:131859323-131859345 | intergenic | 16,319 | 0.27 | 0.00 |
|  |  |  | PTCHD2-RP1-69M21.2-DISP3 | 3 | chr1:11563783-11563805 | intergenic | 84,621 | 1.7 | 0.18 |
|  |  |  | LINC00959 | 3 | chr10:130110736-130110758 | Exon | 50 | 0.3 | 0.014 |
| **SgRNA_6** | 94 | 99 | GADD45B-RNU6-993P | 3 | chr19:2494038-2494060 | intergenic | 17,904 | 0.49 | 0.009 |

**Supplementary Table 2**. sgRNAs specific for KDM4A off-targets and chromosome position.

| **Target gene** | **Sequences** | **Forward/Reverse** |
| --- | --- | --- |
| KDM4A | GAACCTGTGCGCGATTCCTG | FW |
| KDM4A | CCTAGCACTGGGATTCAGAGTT | RW |
| KDM4B | TGACATCAGGCCCTCTTTGT | FW |
| KDM4B | TACCAGGACTTAGGCTCCCC | RW |
| KDM4C | GGAAGACCACGTTTGCATGG | FW |
| KDM4C | GCCTCCTGGGTTATCTTGTCA | RW |
| GAPDH | CCACATCGCTCAGACACCAT | FW |
| GAPDH | GCGCCCAATACGACCAAAT | RW |

**Supplementary Table 3**. List of primer sequences


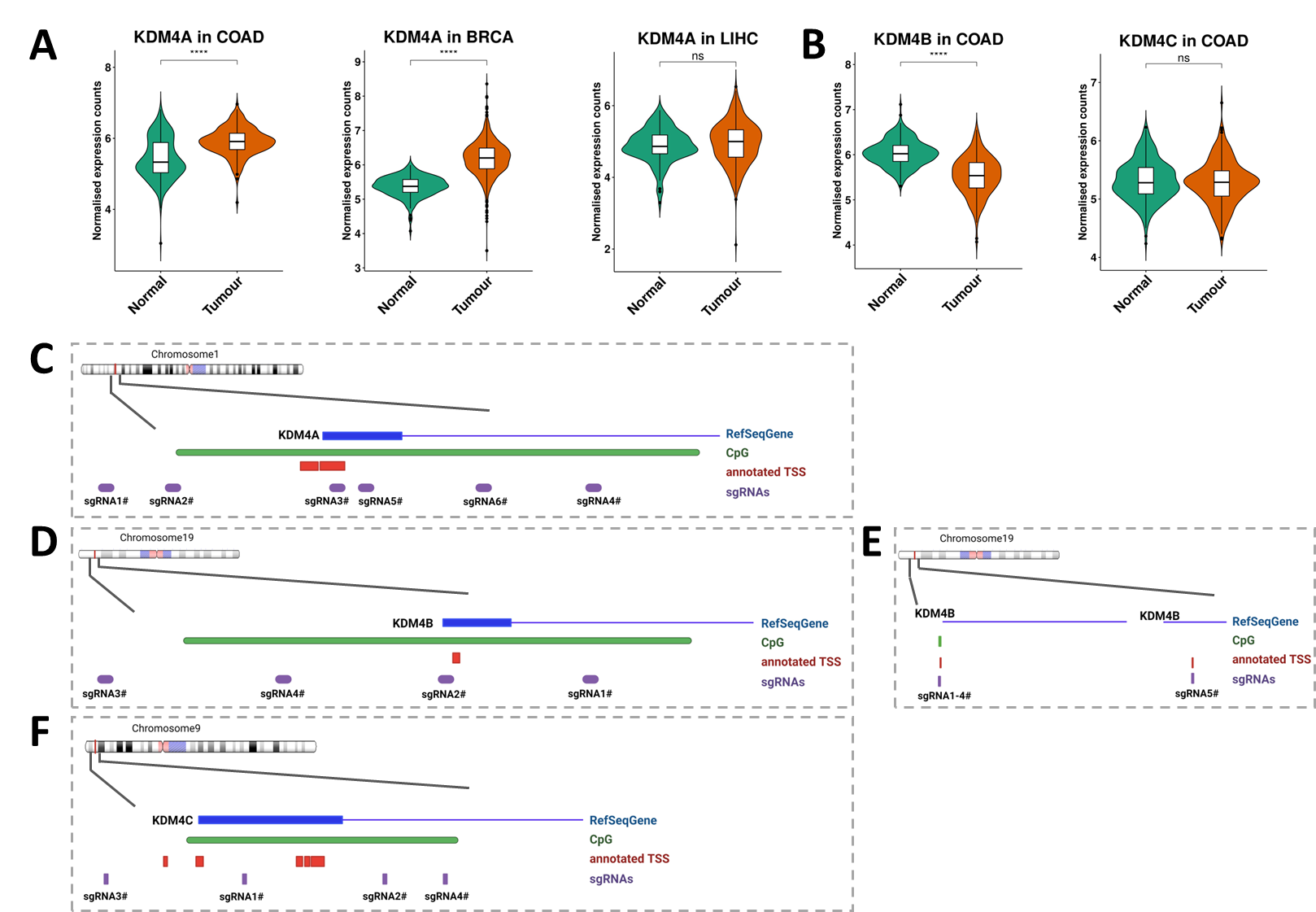


**Supplementary Figure 1.** (**A**) KDM4A gene expression online database in colon adenocarcinoma (COAD), breast invasive carcinoma (BRCA) and liver hepatocellular carcinoma (LIHC) compared to normal samples; (**B**) KDM4B and KDM4C gene expression online database in colon adenocarcinoma (COAD) compared to normal samples; (**C**-**F**) KDM4-A/B/C sgRNAs targeting KDM4A or KDM4B or KDM4C promoter regions. Close to the annotated TSS (in red) and the CpG islands (in green), six sgRNAs were designed for (**C**) KDM4A, five for (**D**-**E**) KDM4B ((**D**) four close the TSS1 (left) and (**E**) one on TSS2 (right)) and (**F**) four for KDM4C. In blue is showed the promoter gene and gene body.

**
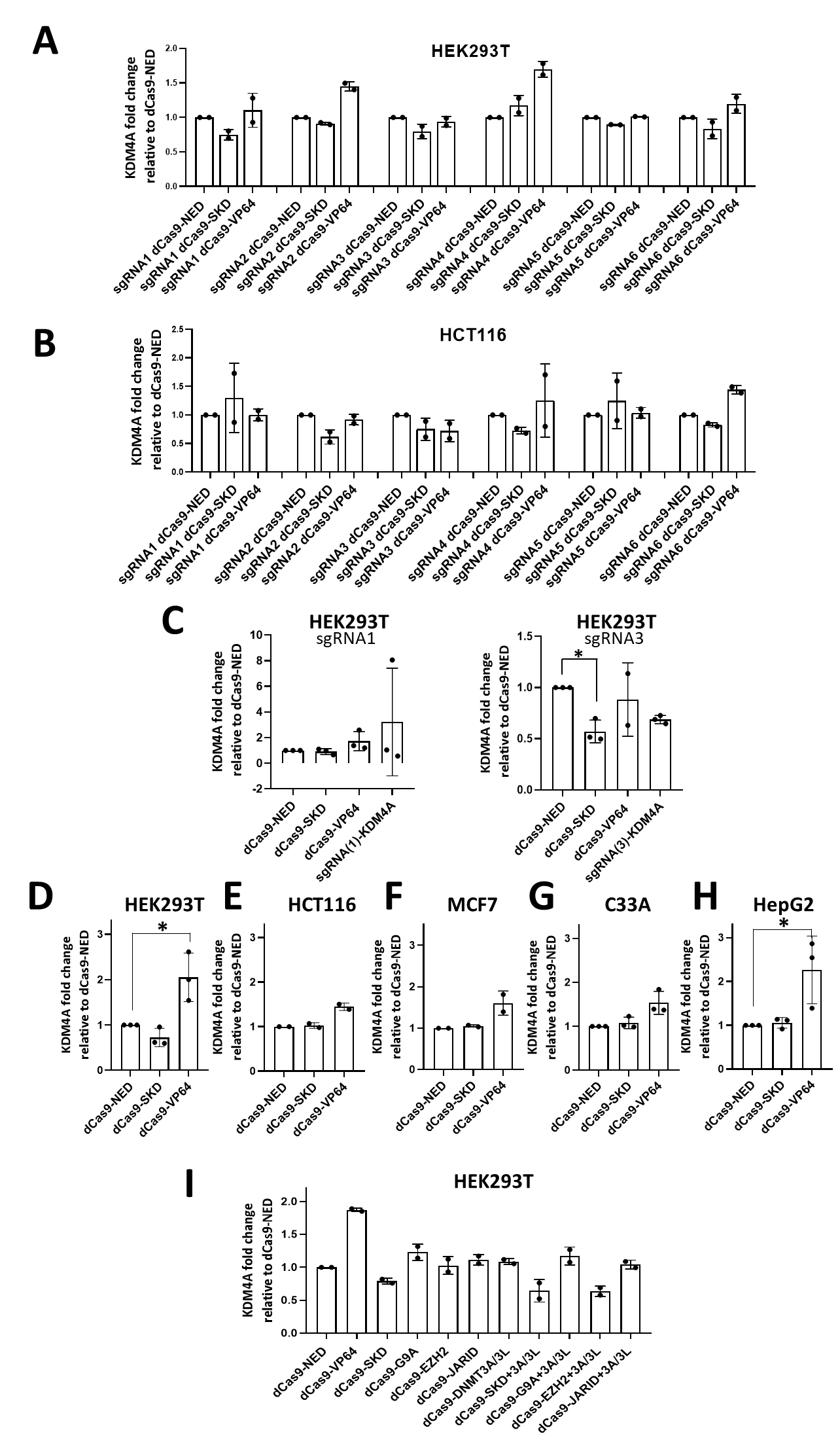
**

**Supplementary Figure 2.** Validation of KDM4A gene expression modulation by epi-editing in (**A**) HEK293T and (**B**) HCT116 using single sgRNA; (**C**) KDM4A gene expression in (**left**) sgRNA(1)-KDM4A and (**right**) sgRNA(3)-KDM4A HEK293T stable cells after 48 h of dCas9-NED, dCas9-SKD, and dCas9-VP64 transient transfected. KDM4A gene expression modulation using mix of sgRNA-KDM4A in (**D**) HEK293T, (**E**) HCT116, (**F**) MCF7, (**G**) C33A and (**H**) HepG2 cells for dCas9-SKD and dCas9-VP64 compared to dCas9 without an effector domain (NED); (**I**) KDM4A gene expression modulation in HEK293T cells, 48 h after transfection, using sgRNA-KDM4A and dCas9-G9A (H3K9me1/me2 writer), or dCas9-EZH2 (H3K27me3 writer), or dCas9-JARID (H3K4me3/me2 eraser), or dCas9-DNMT3A/3L (writer of DNA methylation) alone or in combination.


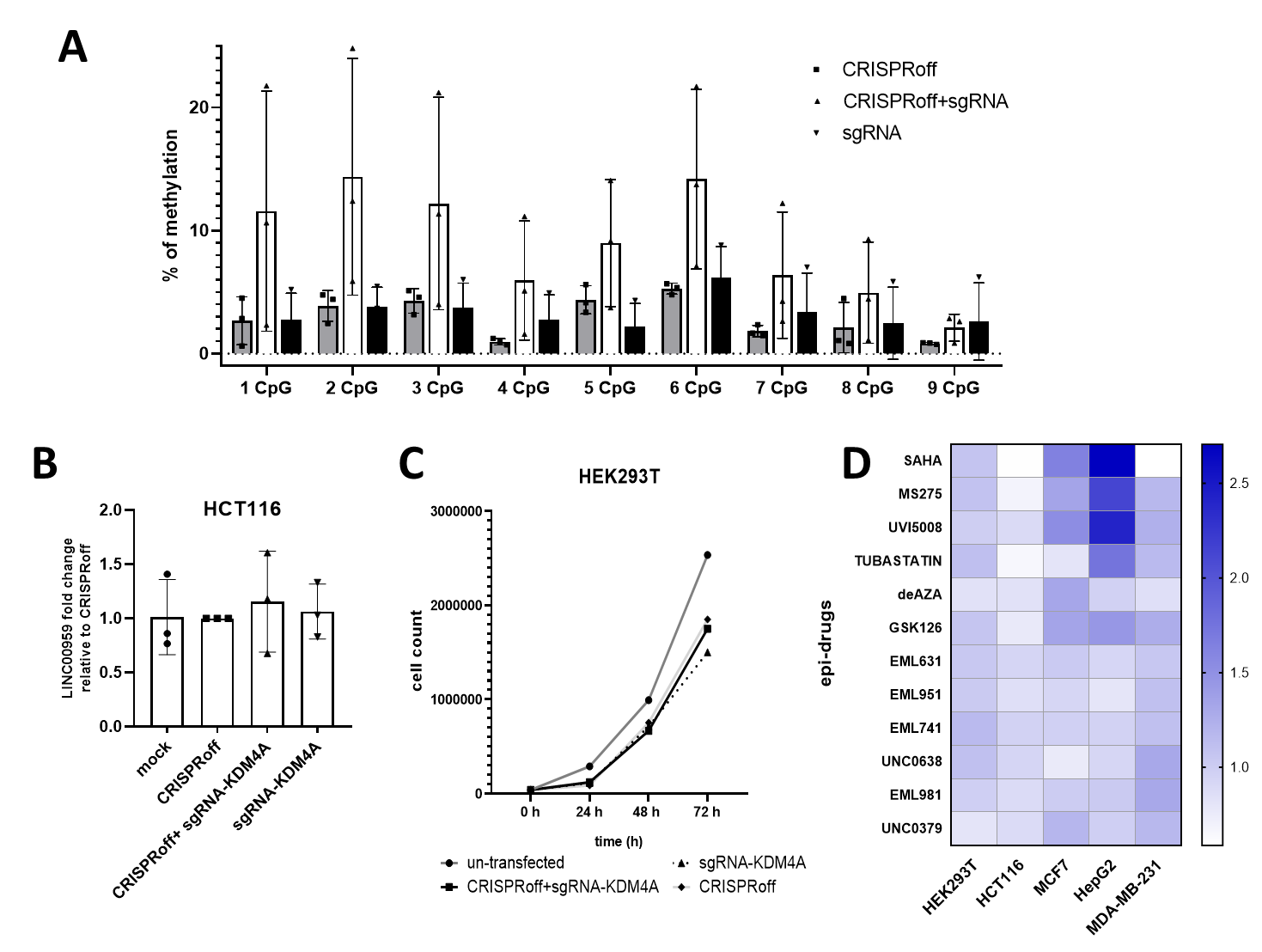


**Supplementary Figure 3.** (**A**) DNA methylation profile of analysed KDM4A promoter region (chr1:43,649,905–43,650,085), in HCT116 cells 3 days post-transfection; (**B**) *LINC00959* gene expression modulation using CRISPRoff+sgRNA-KDM4A. CRISPRoff and sgRNA-KDM4A transfected alone, and mock transfected cells (cells transfected with empty MLM3636) were used as controls; (**C**) Proliferation assay in HEK293T cells upon epi-editing downregulating KDM4A. The dark grey line (symbol: circle) shows the un-transfected cells, the black line (symbol: square) shows the cells transfected with CRISPRoff and sgRNA-KDM4A, in the dashed line (symbol: triangle) the cell transfected only with sgRNA-KDM4A, in light grey (symbol: rhombus) the cells only with CRISPRoff; (**D**) KDM4A expression in HEK293T, HCT116, MCF7, HepG2, and MD-MB-231 cells after treatment with 12 epi-drugs: HDACs inhibitors (SAHA, MS275, UVI5008, and TUBASTATIN); DNMTs inhibitor (deAZA); EZH2 inhibitor (GSK126); Spindlin-1 inhibitor (EML631); MRG15 inhibitor (EML951); G9a inhibitors (EML741, and UNC0638); CARM-1 inhibitor (EML981); SETD8 (UNC0379) (data represent the mean of two independent experiments).


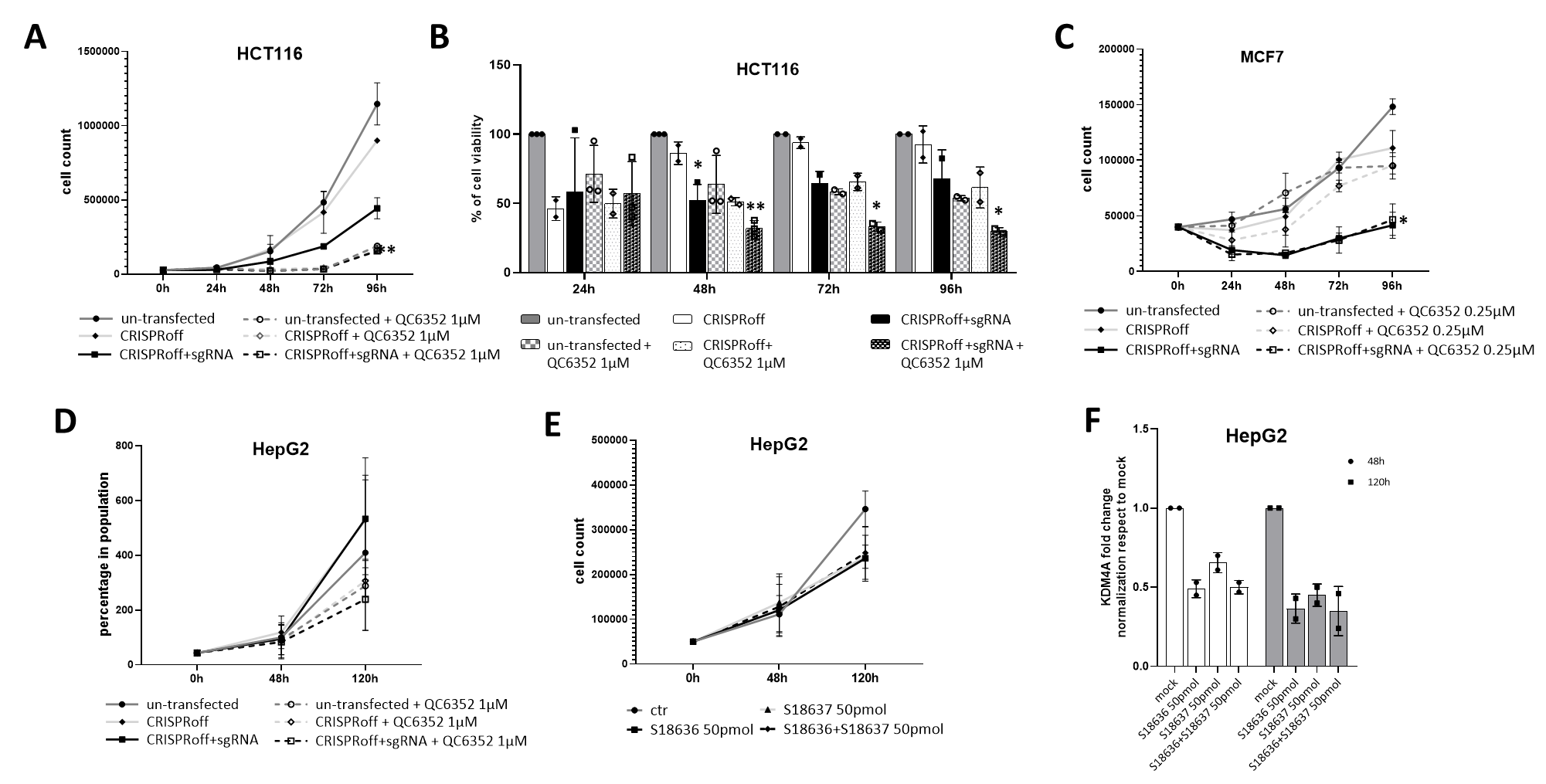


**Supplementary Figure 4.** Proliferation assay in (**A**) HCT116, (**C**) MCF7 and (**D**) HepG2 cells with/without sgRNA-KDM4A (sgRNA) and CRISPRoff treated with QC6352 at (**A**,**D**) 1µM, and (**C**) 0.25µM or DMSO; (**B**) MTS assay in HCT116 with/without sgRNA-KDM4A and CRISPRoff treated with QC6352 at 1µM or DMSO. The dark grey lines and bars show the un-transfected cells (symbol: circle), in light grey the cells only with CRISPRoff (symbol: rhombus), in black the cells transfected with CRISPRoff+ sgRNA-KDM4A (symbol: square). The solid lines and bars represent the cells only transfected with epi-editing, and the dashed lines and bars, the cells treated with QC6352 24h after transfection; (**E**) Proliferation assay in HepG2 transfected with KDM4A or siRNA S18636 target exons 15-16 (black line, symbol square), or siRNA S18637 target exons 19 (light grey line, symbol triangle) or both (black dashed line, symbol circle); (**F**) KDM4A gene expression in HepG2 transfected with or siRNA S18636 or siRNA S18637 or both for 48 h (white) and 120 h (grey).


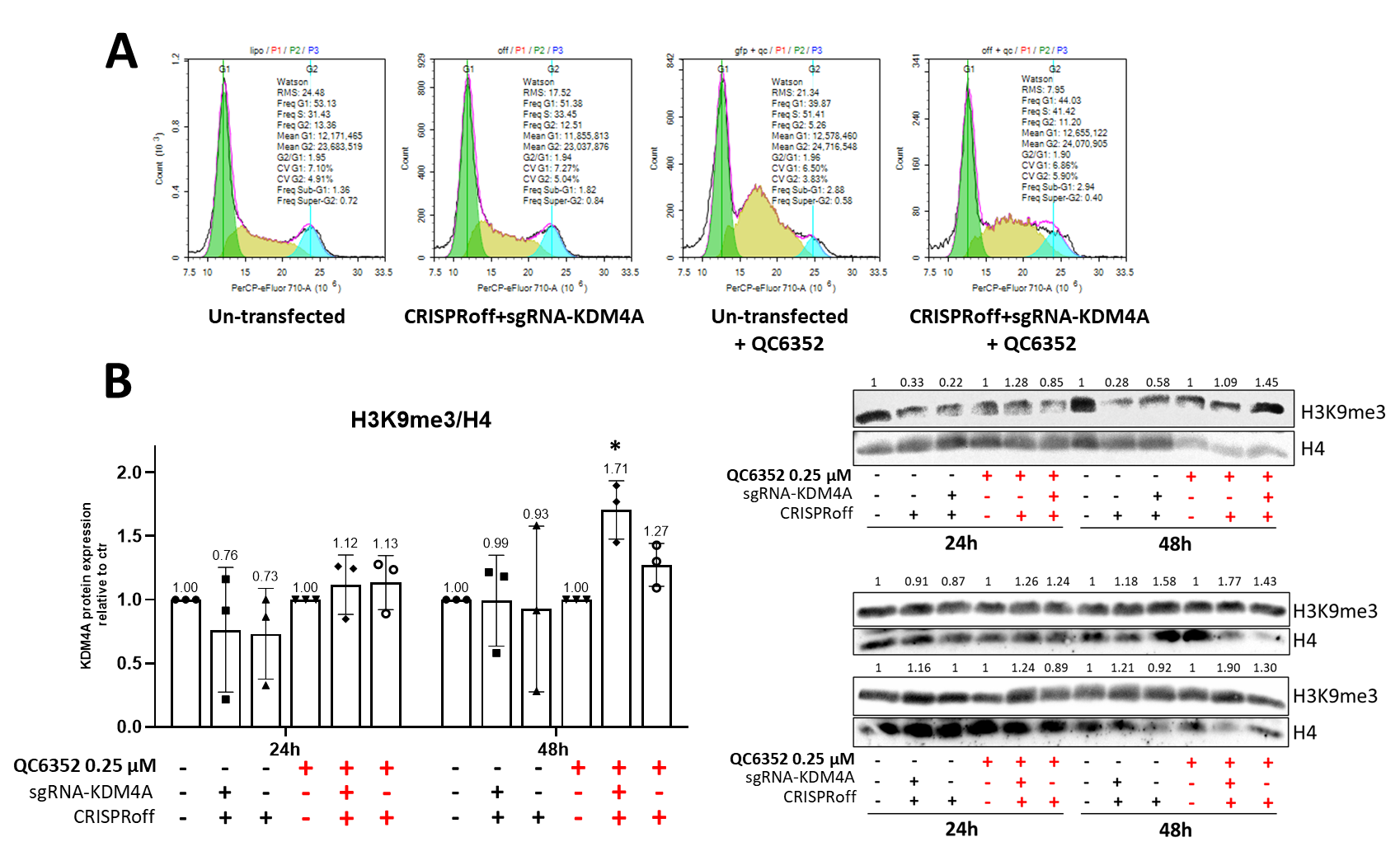


**Supplementary Figure 5.** (**A**) HCT116 cell cycle with/without CRISPRoff+sgRNA-KDM4A treated with QC6352 at 1µM or DMSO; (**B**) Western blot H3K9me3 and H4 in HCT116 with/without CRISPRoff+sgRNA-KDM4A treated with QC6352 at 0.25µM for 24h and 48h or DMSO. On the left, the graph showed the average of blots quantization, obtained with ImageJ.


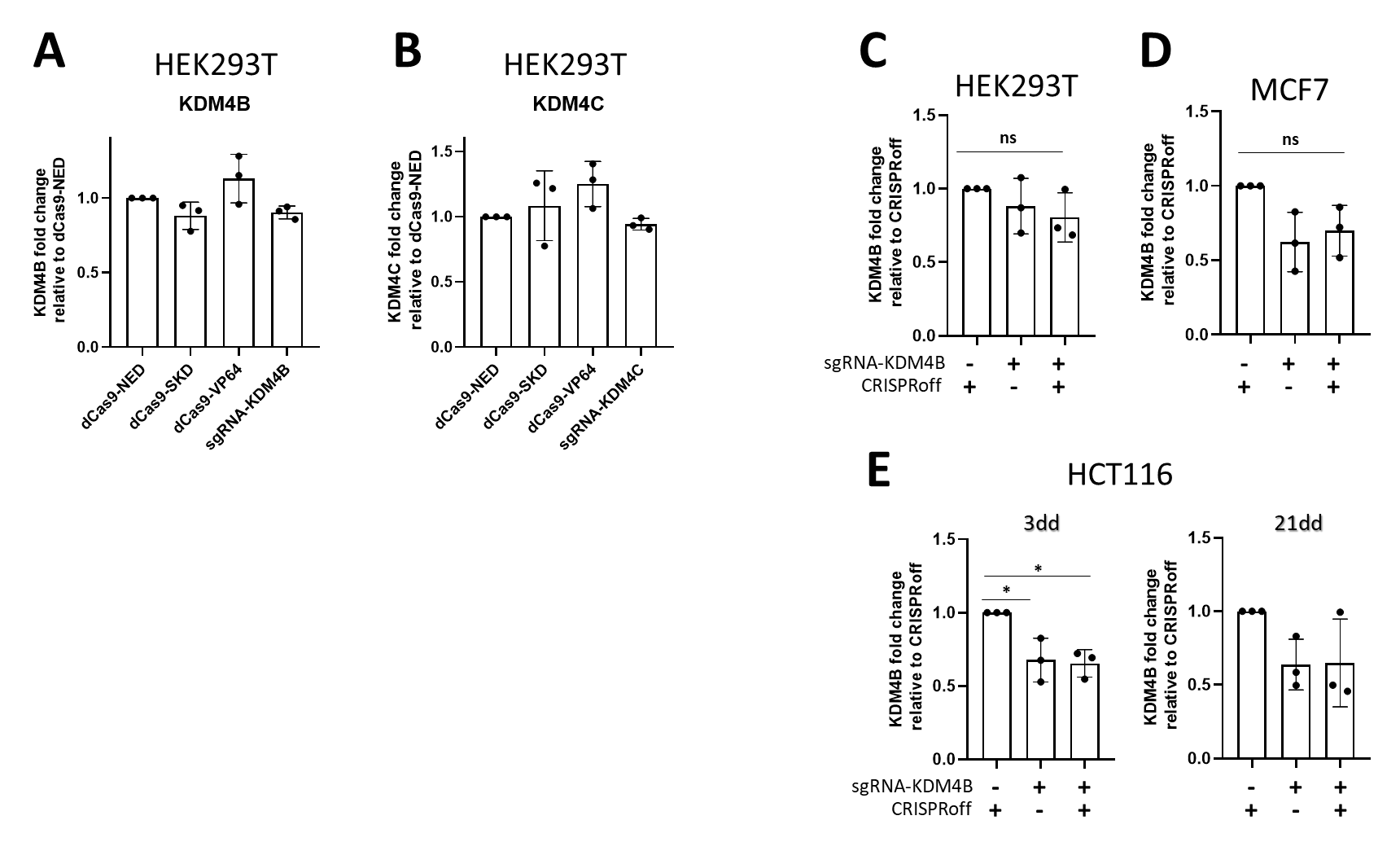


**Supplementary Figure 6.** (**A**) KDM4B and (**B**) KDM4C gene expression modulation by epi-editing in HEK293T 48h after transfection; (**C-D**) KDM4B gene expression reduction by epi-editing in (**C**) HEK293T and (**D**) MCF7 3 days after CRISPRoff + sgRNA-KDM4B transfection; (**E**) KDM4B gene evaluation in HCT116 after 3 and 21 days after CRISPRoff+sgRNA-KDM4B transfection.
